# (*E*)-2,4-Dichloro-6-{1-[(2-chloro­eth­yl)imino]­eth­yl}phenol

**DOI:** 10.1107/S160053681004448X

**Published:** 2010-11-10

**Authors:** Yong-Sheng Xie, Wen-Liang Dong, Li-Ping He, Xin-Ling Zhang, Bao-Xiang Zhao

**Affiliations:** a College of Chemical and Environment Engineering, Chongqing Three Gorges University, Chongqing 404100, People’s Republic of China; b Shandong University of Traditional Chinese Medicine, Jinan 250355, People’s Republic of China; c Editorial Department of College Journal, Chongqing Three Gorges University, Chongqing 404100, People’s Republic of China; d School of Chemistry and Chemical Engineering, Shandong University, Jinan 250100, People’s Republic of China

## Abstract

The title Schiff base compound, C_10_H_10_Cl_3_NO, was prepared by the condensation of 1-(3,5-dichloro-2-hy­droxy­phen­yl)ethanone with chloro­ethyl­amine. The imine adopts an *E* configuration with respect to the C=N bond. The H atom of the phenolic OH group is disordered over two positions with site occupation factors of 0.52 (7) and 0.48 (7), respectively, and the major occupancy component is involved in an intramolecular N—H⋯O hydrogen bond. The compound therefore exists in an iminium–phenolate as well as in the imino–phenol form. In the crystal, mol­ecules are connected by C—H⋯O and C—H⋯Cl hydrogen bonds and Cl⋯Cl inter­actions [3.7864 (9) Å] into a three-dimensional network. In addition, inter­molecular π–π stacking inter­actions [centroid–centroid distance = 4.4312 (9) Å] are observed.

## Related literature

For a related structure, see: Wang *et al.* (2010 ▶). For applications of Schiff base ligands, see: Yin *et al.* (2004 ▶); Böhme & Günther (2007 ▶).
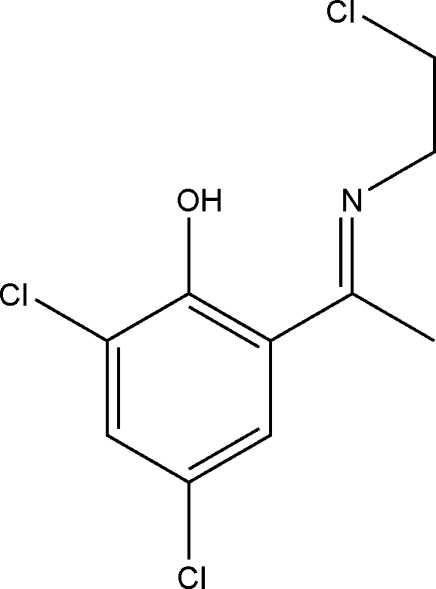


## Experimental

### 

#### Crystal data

C_10_H_10_Cl_3_NO
*M* *_r_* = 266.54
Monoclinic, 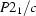

*a* = 14.5710 (2) Å
*b* = 10.2323 (2) Å
*c* = 7.7384 (1) Å
β = 94.376 (1)°
*V* = 1150.39 (3) Å^3^
*Z* = 4
Mo *K*α radiation
μ = 0.77 mm^−1^
*T* = 296 K
0.38 × 0.19 × 0.07 mm

#### Data collection

Bruker APEXII CCD area-detector diffractometer
Absorption correction: multi-scan (*SADABS*; Bruker, 2005 ▶) *T*_min_ = 0.762, *T*_max_ = 0.951
8253 measured reflections
2625 independent reflections
1940 reflections with *I* > 2σ(*I*)
*R*_int_ = 0.023

#### Refinement

*R*[*F* ^2^ > 2σ(*F* ^2^)] = 0.033
*wR*(*F* ^2^) = 0.091
*S* = 1.02
2625 reflections
146 parameters
2 restraints
H atoms treated by a mixture of independent and constrained refinement
Δρ_max_ = 0.25 e Å^−3^
Δρ_min_ = −0.21 e Å^−3^

### 

Data collection: *APEX2* (Bruker, 2005 ▶); cell refinement: *SAINT* (Bruker, 2005 ▶); data reduction: *SAINT*; program(s) used to solve structure: *SIR97* (Altomare *et al.*, 1999 ▶); program(s) used to refine structure: *SHELXL97* (Sheldrick, 2008 ▶); molecular graphics: *SHELXTL* (Sheldrick, 2008 ▶); software used to prepare material for publication: *publCIF* (Westrip, 2010 ▶).

## Supplementary Material

Crystal structure: contains datablocks I, global. DOI: 10.1107/S160053681004448X/im2238sup1.cif

Structure factors: contains datablocks I. DOI: 10.1107/S160053681004448X/im2238Isup2.hkl

Additional supplementary materials:  crystallographic information; 3D view; checkCIF report

## Figures and Tables

**Table 1:** Hydrogen-bond geometry (Å, °)

*D*—H⋯*A*	*D*—H	H⋯*A*	*D*⋯*A*	*D*—H⋯*A*
N1—H1*N*⋯O1	0.88 (2)	1.68 (2)	2.479 (2)	150 (4)
C10—H10*B*⋯O1^i^	0.97	2.48	3.416 (2)	159
C10—H10*A*⋯Cl3^ii^	0.97	2.86	3.621 (2)	136

Symmetry codes: (i) 
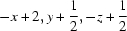
; (ii) 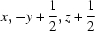.

**Fig. 1.**
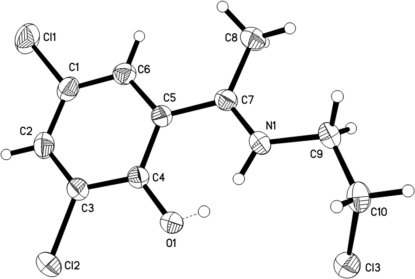
Molecular structure of the title compound, showing the atom-numbering scheme. Displacement ellipsoids are drawn at the 30% probability level.

**Fig. 2.**
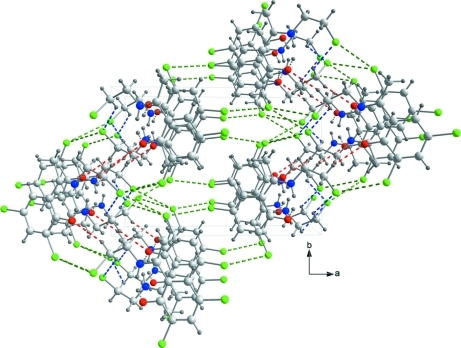
Crystal packing of title compound viewed along the b axis. C—H···O and C—H···Cl hydrogen bonds are displayed as red and blue dashed lines, respectively. Cl···Cl interactions are shown as green dashed lines.

**Fig. 3.**
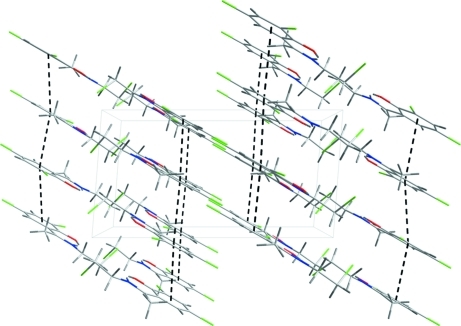
Crystal packing of title compound viewed along the c axis. The π–π interactions are shown as black dashed lines.

## supplementary crystallographic information

### Comment

Schiff-base ligands have attracted much attention over the years, e.g. as ligands in organotin(IV) compounds owing to their anti-tumour activities (Yin *et al.*, 2004), and in silicon complexes applied to the field of photovoltaic applications, as coloring material and due to their antimicrobial activity. (Böhme & Günther, 2007). We report here the crystal structure of the title Schiff-base ligand (Fig. 1).

The molecular structure of the ligand is represented in Fig. 1. The bond lengths and angles are in eligible range. The C7—N1 and C9—N1 bond lengths of 1.290 (2), 1.460 (2) Å, respectively, conform to the value for a double and single bonds and they are comparable with the corresponding bond lengths in similar Schiff-base compounds (Wang *et al.*, 2010). The hydrogen atom of the phenolic OH group is disordered over two positions with site occupation factors of 0.52 and 0.48, respectively. The compound therefore exists in an iminium-phenolate as well as in an imino-phenol form. The situation may be interpreted as the intramolecular protonation of the basic imine nitrogen by the acidic phenol group. In the crystal, molecules are connected by C—H···O, C—H···Cl (Table 1) and Cl···Cl interactions [Cl1···Cl2 distance = 3.7864 (9) Å for symmetry operation -*x* + 1, -*y* + 1, -*z*; Cl2···Cl3 distance = 3.7709 (7) Å for symmetry operation -*x* + 2, *y* - 1/2, -*z* + 1/2; Cl2···Cl3 distance = 3.7789 (8) Å for symmetry operation -*x* + 2, -*y* + 1, -*z*] into a network (Fig. 2). In addition, weak intermolecular π–π interactions serve to stabilize the extended structure [*Cg*···*Cg* distance = 4.4312 (9) Å for symmetry operation *x*, -*y* + 1/2, *z* - 1/2 and *x*, -*y* + 1/2, *z* + 1/2 (The *Cg* is the centroid of the phenyl ring)].

### Experimental

To a mixture of 1-(3,5-dichloro-2-hydroxy-phenyl)-ethanone (5.1 g, 25 mmol) and chloroethylamine hydrochloride (5.8 g, 50 mmol) in ethanol (150 ml) was added triethylamine (5.1 g, 50 mmol). The mixture was heated to reflux for 10 min. After being cooled to room temperature, the resulting precipitate was filtrated and washed with water to afford the product, *E*-2,4-dichloro-6-(1-(2-chloroethylimino)ethyl)phenol in 84% yield. Crystals of the title compound suitable for X-ray diffraction were obtained by slow evaporation of a solution of the solid in ethyl acetate at room temperature for 7 d.

### Refinement

All H atoms at carbon were placed geometrically and refined using a riding model with C—H = 0.97 Å (for CH_2_ group), 0.96 Å (for CH_3_ group) and 0.93 Å (for aryl H atoms). The isotropic atomic displacement parameters of hydrogen atoms were set to 1.5 × *Ueq* (CH_3_) and 1.2 × *Ueq* (CH_2_, C_ar_H) of the parent atoms. Positions of hydrogen atoms at N1 and O1 were taken from difference Fourier maps and were refined using PART instructions.

### Crystal data

**Table:**

C_10_H_10_Cl_3_NO	*F*(000) = 544
*M**_r_* = 266.54	*D*_x_ = 1.539 Mg m^−^^3^
Monoclinic, *P*2_1_/*c*	Mo *K*α radiation, λ = 0.71073 Å
Hall symbol: -P 2ybc	Cell parameters from 2504 reflections
*a* = 14.5710 (2) Å	θ = 2.4–25.9°
*b* = 10.2323 (2) Å	µ = 0.77 mm^−^^1^
*c* = 7.7384 (1) Å	*T* = 296 K
β = 94.376 (1)°	Plate, orange
*V* = 1150.39 (3) Å^3^	0.38 × 0.19 × 0.07 mm
*Z* = 4	

### Data collection

**Table:**

Bruker APEXII CCD area-detector diffractometer	2625 independent reflections
Radiation source: fine-focus sealed tube	1940 reflections with *I* > 2σ(*I*)
graphite	*R*_int_ = 0.023
φ and ω scans	θ_max_ = 27.5°, θ_min_ = 2.4°
Absorption correction: multi-scan (*SADABS*; Bruker, 2005)	*h* = −18→17
*T*_min_ = 0.762, *T*_max_ = 0.951	*k* = −10→13
8253 measured reflections	*l* = −8→10

### Refinement

**Table:**

Refinement on *F*^2^	Primary atom site location: structure-invariant direct methods
Least-squares matrix: full	Secondary atom site location: difference Fourier map
*R*[*F*^2^ > 2σ(*F*^2^)] = 0.033	Hydrogen site location: inferred from neighbouring sites
*wR*(*F*^2^) = 0.091	H atoms treated by a mixture of independent and constrained refinement
*S* = 1.01	*w* = 1/[σ^2^(*F*_o_^2^) + (0.0452*P*)^2^ + 0.1559*P*] where *P* = (*F*_o_^2^ + 2*F*_c_^2^)/3
2625 reflections	(Δ/σ)_max_ = 0.006
146 parameters	Δρ_max_ = 0.25 e Å^−^^3^
2 restraints	Δρ_min_ = −0.21 e Å^−^^3^

### Special details

**Table:**

Geometry. All e.s.d.'s (except the e.s.d. in the dihedral angle between two l.s. planes) are estimated using the full covariance matrix. The cell e.s.d.'s are taken into account individually in the estimation of e.s.d.'s in distances, angles and torsion angles; correlations between e.s.d.'s in cell parameters are only used when they are defined by crystal symmetry. An approximate (isotropic) treatment of cell e.s.d.'s is used for estimating e.s.d.'s involving l.s. planes.
Refinement. Refinement of *F*^2^ against ALL reflections. The weighted *R*-factor *wR* and goodness of fit *S* are based on *F*^2^, conventional *R*-factors *R* are based on *F*, with *F* set to zero for negative *F*^2^. The threshold expression of *F*^2^ > σ(*F*^2^) is used only for calculating *R*-factors(gt) *etc*. and is not relevant to the choice of reflections for refinement. *R*-factors based on *F*^2^ are statistically about twice as large as those based on *F*, and *R*- factors based on ALL data will be even larger.

### Fractional atomic coordinates and isotropic or equivalent isotropic displacement parameters (Å^2^)

**Table:**

	*x*	*y*	*z*	*U*_iso_*/*U*_eq_	Occ. (<1)
C1	0.57736 (13)	0.15817 (19)	−0.1505 (2)	0.0498 (4)	
C2	0.61146 (13)	0.0340 (2)	−0.1143 (2)	0.0516 (5)	
H2	0.5771	−0.0395	−0.1481	0.062*	
C3	0.69659 (12)	0.02119 (17)	−0.0279 (2)	0.0455 (4)	
C4	0.75065 (12)	0.12988 (16)	0.0289 (2)	0.0401 (4)	
C5	0.71381 (12)	0.25627 (16)	−0.0117 (2)	0.0388 (4)	
C6	0.62665 (12)	0.26726 (18)	−0.1027 (2)	0.0458 (4)	
H6	0.6026	0.3495	−0.1303	0.055*	
C7	0.76729 (12)	0.37256 (16)	0.0405 (2)	0.0403 (4)	
C8	0.73432 (14)	0.50736 (18)	−0.0072 (3)	0.0559 (5)	
H8A	0.6689	0.5122	0.0002	0.084*	
H8B	0.7487	0.5268	−0.1235	0.084*	
H8C	0.7642	0.5696	0.0710	0.084*	
C9	0.90715 (13)	0.45820 (17)	0.1910 (2)	0.0491 (4)	
H9A	0.8749	0.5189	0.2611	0.059*	
H9B	0.9276	0.5057	0.0925	0.059*	
C10	0.98893 (13)	0.40380 (19)	0.2962 (2)	0.0509 (5)	
H10A	0.9682	0.3582	0.3961	0.061*	
H10B	1.0284	0.4752	0.3382	0.061*	
Cl1	0.46922 (4)	0.17442 (7)	−0.26165 (8)	0.0791 (2)	
Cl2	0.74236 (4)	−0.13318 (4)	0.01161 (8)	0.06643 (18)	
Cl3	1.05345 (3)	0.29415 (5)	0.17400 (7)	0.05817 (16)	
N1	0.84464 (10)	0.35378 (14)	0.12961 (19)	0.0418 (3)	
H1N	0.858 (3)	0.271 (2)	0.146 (5)	0.049 (16)*	0.52 (7)
H1O	0.855 (3)	0.183 (3)	0.145 (6)	0.07 (2)*	0.48 (7)
O1	0.83069 (9)	0.11256 (12)	0.11379 (18)	0.0494 (3)	

### Atomic displacement parameters (Å^2^)

**Table:**

	*U*^11^	*U*^22^	*U*^33^	*U*^12^	*U*^13^	*U*^23^
C1	0.0381 (10)	0.0583 (11)	0.0523 (10)	−0.0039 (9)	−0.0005 (8)	0.0049 (9)
C2	0.0472 (11)	0.0491 (10)	0.0583 (11)	−0.0119 (9)	0.0041 (9)	−0.0039 (9)
C3	0.0451 (10)	0.0370 (9)	0.0551 (10)	−0.0010 (8)	0.0077 (8)	−0.0016 (7)
C4	0.0392 (10)	0.0368 (8)	0.0450 (9)	−0.0005 (7)	0.0069 (7)	−0.0006 (7)
C5	0.0387 (9)	0.0364 (8)	0.0417 (9)	−0.0005 (7)	0.0051 (7)	0.0023 (7)
C6	0.0421 (10)	0.0449 (10)	0.0506 (10)	0.0042 (8)	0.0041 (8)	0.0077 (8)
C7	0.0428 (10)	0.0360 (8)	0.0428 (9)	0.0027 (7)	0.0081 (7)	0.0028 (7)
C8	0.0577 (12)	0.0382 (9)	0.0709 (12)	0.0041 (9)	−0.0014 (10)	0.0076 (9)
C9	0.0515 (11)	0.0348 (8)	0.0610 (11)	−0.0068 (8)	0.0040 (9)	−0.0030 (8)
C10	0.0561 (12)	0.0511 (10)	0.0450 (9)	−0.0121 (9)	0.0002 (8)	−0.0071 (8)
Cl1	0.0484 (3)	0.0859 (4)	0.0987 (4)	−0.0121 (3)	−0.0218 (3)	0.0179 (3)
Cl2	0.0629 (4)	0.0339 (2)	0.1020 (4)	0.0003 (2)	0.0030 (3)	−0.0027 (2)
Cl3	0.0544 (3)	0.0519 (3)	0.0673 (3)	0.0025 (2)	−0.0016 (2)	−0.0059 (2)
N1	0.0448 (9)	0.0310 (7)	0.0493 (8)	−0.0020 (6)	0.0019 (7)	−0.0003 (6)
O1	0.0408 (7)	0.0350 (7)	0.0710 (8)	0.0018 (6)	−0.0055 (6)	0.0007 (6)

### Geometric parameters (Å, °)

**Table:**

C1—C6	1.363 (3)	C7—C8	1.498 (2)
C1—C2	1.385 (3)	C8—H8A	0.9600
C1—Cl1	1.7446 (19)	C8—H8B	0.9600
C2—C3	1.370 (2)	C8—H8C	0.9600
C2—H2	0.9300	C9—N1	1.460 (2)
C3—C4	1.413 (2)	C9—C10	1.498 (3)
C3—Cl2	1.7326 (18)	C9—H9A	0.9700
C4—O1	1.306 (2)	C9—H9B	0.9700
C4—C5	1.426 (2)	C10—Cl3	1.781 (2)
C5—C6	1.409 (2)	C10—H10A	0.9700
C5—C7	1.462 (2)	C10—H10B	0.9700
C6—H6	0.9300	N1—H1N	0.874 (19)
C7—N1	1.290 (2)	O1—H1O	0.827 (19)
			
C6—C1—C2	121.53 (17)	C7—C8—H8B	109.5
C6—C1—Cl1	119.55 (15)	H8A—C8—H8B	109.5
C2—C1—Cl1	118.92 (15)	C7—C8—H8C	109.5
C3—C2—C1	118.94 (17)	H8A—C8—H8C	109.5
C3—C2—H2	120.5	H8B—C8—H8C	109.5
C1—C2—H2	120.5	N1—C9—C10	110.83 (15)
C2—C3—C4	122.61 (16)	N1—C9—H9A	109.5
C2—C3—Cl2	119.69 (14)	C10—C9—H9A	109.5
C4—C3—Cl2	117.69 (13)	N1—C9—H9B	109.5
O1—C4—C3	120.31 (15)	C10—C9—H9B	109.5
O1—C4—C5	122.71 (15)	H9A—C9—H9B	108.1
C3—C4—C5	116.98 (15)	C9—C10—Cl3	112.06 (12)
C6—C5—C4	119.49 (16)	C9—C10—H10A	109.2
C6—C5—C7	120.94 (15)	Cl3—C10—H10A	109.2
C4—C5—C7	119.56 (15)	C9—C10—H10B	109.2
C1—C6—C5	120.43 (17)	Cl3—C10—H10B	109.2
C1—C6—H6	119.8	H10A—C10—H10B	107.9
C5—C6—H6	119.8	C7—N1—C9	124.26 (15)
N1—C7—C5	116.87 (14)	C7—N1—H1N	113 (3)
N1—C7—C8	121.32 (16)	C9—N1—H1N	122 (3)
C5—C7—C8	121.82 (16)	C4—O1—H1O	112 (4)
C7—C8—H8A	109.5		
			
C6—C1—C2—C3	0.2 (3)	C2—C1—C6—C5	−1.1 (3)
Cl1—C1—C2—C3	179.64 (14)	Cl1—C1—C6—C5	179.53 (13)
C1—C2—C3—C4	1.1 (3)	C4—C5—C6—C1	0.5 (3)
C1—C2—C3—Cl2	−177.60 (14)	C7—C5—C6—C1	−179.91 (16)
C2—C3—C4—O1	178.66 (16)	C6—C5—C7—N1	177.17 (15)
Cl2—C3—C4—O1	−2.6 (2)	C4—C5—C7—N1	−3.3 (2)
C2—C3—C4—C5	−1.6 (3)	C6—C5—C7—C8	−3.0 (3)
Cl2—C3—C4—C5	177.17 (12)	C4—C5—C7—C8	176.56 (16)
O1—C4—C5—C6	−179.54 (15)	N1—C9—C10—Cl3	60.56 (18)
C3—C4—C5—C6	0.7 (2)	C5—C7—N1—C9	179.36 (15)
O1—C4—C5—C7	0.9 (3)	C8—C7—N1—C9	−0.5 (3)
C3—C4—C5—C7	−178.81 (15)	C10—C9—N1—C7	177.78 (16)

### Hydrogen-bond geometry (Å, °)

**Table:**

*D*—H···*A*	*D*—H	H···*A*	*D*···*A*	*D*—H···*A*
N1—H1N···O1	0.88 (2)	1.68 (2)	2.479 (2)	150 (4)
C10—H10B···O1^i^	0.97	2.48	3.416 (2)	159
C10—H10A···Cl3^ii^	0.97	2.86	3.621 (2)	136

Symmetry codes: (i) −*x*+2, *y*+1/2, −*z*+1/2; (ii) *x*, −*y*+1/2, *z*+1/2.

## References

[bb1] Altomare, A., Burla, M. C., Camalli, M., Cascarano, G. L., Giacovazzo, C., Guagliardi, A., Moliterni, A. G. G., Polidori, G. & Spagna, R. (1999). *J. Appl. Cryst.* **32**, 115–119.

[bb2] Böhme, U. & Günther, B. (2007). *Inorg. Chem. Commun.* **10**, 482-484.

[bb3] Bruker (2005). *APEX2*, *SAINT* and *SADABS* Bruker AXS Inc., Madison, Wisconsin, USA.

[bb4] Sheldrick, G. M. (2008). *Acta Cryst.* A**64**, 112–122. 10.1107/S0108767307043930 18156677

[bb5] Wang, C.-H., Liu, Y.-C., Lin, C.-H. & Ko, B.-T. (2010). *Acta Cryst.* E**66**, o745. 10.1107/S1600536810007610 PMC2983772 21580590

[bb6] Westrip, S. P. (2010). *J. Appl. Cryst.* **43**, 920–925.

[bb7] Yin, H. D., Wang, Q. B. & Xue, S. C. (2004). *J. Organomet. Chem.* **689**, 2480–2485.

